# Low low-density lipoprotein (LDL), cholesterol and triglycerides plasma levels are associated with reduced risk of arterial occlusive events in chronic myeloid leukemia patients treated with ponatinib in the real-life. A *Campus CML* study

**DOI:** 10.1038/s41408-020-0333-2

**Published:** 2020-06-08

**Authors:** Giovanni Caocci, Olga Mulas, Isabella Capodanno, Elisabetta Abruzzese, Alessandra Iurlo, Luigiana Luciano, Francesco Albano, Mario Annunziata, Mario Tiribelli, Massimiliano Bonifacio, Sara Galimberti, Fausto Castagnetti, Nicola Sgherza, Fabio Stagno, Antonella Gozzini, Ester Maria Orlandi, Debora Luzi, Gianni Binotto, Patrizia Pregno, Claudio Fozza, Fabio Efficace, Maria Pina Simula, Malgorzata Monika Trawinska, Daniele Cattaneo, Fiorenza De Gregorio, Immacolata Attolico, Rossella Stella, Luigi Scaffidi, Claudia Baratè, Gabriele Gugliotta, Emilia Scalzulli, Chiara Elena, Francesca Pirillo, Robin Foà, Massimo Breccia, Giorgio La Nasa

**Affiliations:** 1 Hematology Unit, Businco Hospital, Department of Medical Sciences and Public Health, University of Cagliari, Cagliari, Italy; 2 Hematology Unit, Azienda Unità Sanitaria Locale-IRCCS, Reggio Emilia, Italy; 3 Hematology Unit, Sant’Eugenio Hospital Tor Vergata University, Rome, Italy; 4 Hematology Unit, Fondazione IRCCS Ca’ Granda Ospedale Maggiore Policlinico, Milano, Italy; 5 Hematology Unit “Federico II” University of Naples, Naples, Italy; 6 Department of Emergency and Organ Transplantation – Hematology Section, University of Bari, Bari, Italy; 7 Hematology Unit, Cardarelli Hospital, Naples, Italy; 8 Division of Hematology and BMT, University of Udine, Udine, Italy; 9 Department of Medicine, Section of Hematology, University of Verona, Verona, Italy; 10 Department of Clinical and Experimental Medicine, Section of Hematology, University of Pisa, Pisa, Italy; 11 Department of Experimental, Diagnostic and Specialty Medicine, S. Orsola-Malpighi Hospital, University of Bologna, Bologna, Italy; 12 Hematology and Transplant Center, Casa Sollievo della Sofferenza Hospital, San Giovanni Rotondo, Italy; 13 Hematology Unit, AOU Policlinico-V. Emanuele, Rodolico Hospital, Catania, Italy; 14 Hematology Unit, AOU Careggi, University of Florence, Florence, Italy; 15 Division of Hematology, Fondazione IRCCS Policlinico S. Matteo, Pavia, Italy; 16 Division of Hematology, Hematology Unit, Santa Maria Hospital, Terni, Italy; 17 Hematology Unit, University of Padova, Padua, Italy; 18 Hematology Unit, Azienda Ospedaliero-Universitaria Città della Salute e della Scienza, Torino, Italy; 19 Department of Medical, Surgical and Experimental Sciences, University of Sassari, Sassari, Italy; 20 Data Center and Health Outcomes Research Unit, Italian Group for Adult Hematologic Diseases (GIMEMA), Rome, Italy; 21 Hematology, Department of Translational and Precision Medicine, Sapienza University, Policlinico Umberto 1, Rome, Italy

**Keywords:** Risk factors, Myeloproliferative disease

## To the Editor,

Treatment with ponatinib may induce arterial occlusive events (AOEs) that can limit its effectiveness, suggesting caution in its use in patients with pre-existing cardiovascular (CV) risk factors or CV disease. Variable rates of AOEs have been reported in both randomized clinical trials and in real-life studies^1–3^. Several risk factors associated with the development of AOEs have been suggested^4^. In addition, the Systematic Coronary Risk Evaluation (SCORE) scoring system based on sex, age, systolic pressure, smoking and total cholesterol level has been proposed to predict the risk of fatal CV disease over a 10 years period^5,6^. Dyslipidemia is considered a major risk factor for CV disease. New evidences have shown that the atherosclerotic plaque formation is initiated by the accumulation in the arterial wall of low-density lipoproteins (LDL), cholesterol and other cholesterol-rich apolipoprotein (Apo) B-containing lipoproteins^7^. Very recently, the European Society of Cardiology (ESC) and the European Atherosclerosis Society (EAS) have published new guidelines for the management of dyslipidemia and lipid modification in order to reduce the risk of CV events^8^. So far, no data on the management of dyslipidemia in CML patients treated with ponatinib have been reported. We therefore analyzed a large real-life cohort of Italian patients with CML treated with ponatinib outside of clinical trials and evaluated the role of dyslipidemia. The primary endpoint was to establish the association between AOEs and plasma lipoproteins levels; moreover, we estimated the prognostic value of the new SCORE chart to predict AOEs.

A large series of 116 adult CML patients treated with ponatinib in the real life between January 2012 and December 2019 in 20 Italian centers, was investigated. The study was conducted in accordance with the Declaration of Helsinki.

The concentration of plasma cholesterol, HDL, LDL, and triglycerides at CML diagnosis was collected, before starting the treatment with ponatinib and therefore after 3, 6, and 12 months of treatment. Normal plasma levels where considered as following: cholesterol and triglycerides <200 mg/dL, HDL > 40 mg/dL, and LDL < 130 mg/dL. Two additional optimal targets of LDL values for high (<70 mg/dL) and very-high risk CV patients (<55 mg/dL) were also considered in the analysis^8^.

We estimated the new low-risk SCORE chart for European populations^8^. The new version of the SCORE differs slightly from that of 2016^5^, because age has been extended and the interaction between age and the other risk factors has been incorporated, thus reducing the overestimation of risk in older persons. The patients were stratified into low to moderate (SCORE ≤ 5%) or high to very high (SCORE risk >5%) CV risk. We recorded any cases of primary or secondary antithrombotic prophylaxis and treatments of dyslipidemia with statins or fibrates. All AOEs (cerebrovascular, peripheral vascular, and CV events excluding hypertension) were collected. The cumulative incidence of AOEs was estimated after administering ponatinib. The log-rank test was used to compare two or more groups of stratified patients. Multivariate analyses were performed using the Cox proportional hazards regression model. A *p*-value < 0.05 was considered statistically significant.

The patients’ characteristics of the 116 chronic CML patients are shown in Table 1. The median age was 49 years (range 23–81) and the Sokal score was intermediate-high in 76% of patients. A positive history for CV diseases was reported in 20% of patients. The median follow-up was 3.5 years (range 1–5). In the majority of cases (64%), ponatinib was administered as third or subsequent lines of treatment for inefficacy (82%) or intolerance (18%) to other TKIs; at the start of treatment 23% of patients harbored a T315I mutation.

**Table 1:** Characteristics of patients and cardiovascular profile of 116 CML patients treated with ponatinib.

	*N* (%)		*N* (%)
*Sex*		*CVD risk factors*	
Male	67 (58)	Hypertension	26 (22)
Female	49 (42)	Dyslipidemia	41 (35)
*Age at diagnosis*, mean years (range)	49 (23–81)	Obesity (BMI > 24.5)	17 (15)
*Median follow-up*, mean years (range)	3.5 (1–5)	Severe renal insufficiency	1 (1)
*Leukocyte* ×10^3^/µL, mean value (range)	132 (7–515)	Diabetes	13 (11)
*Hemoglobin* g/dL, mean value (range)	12.3 (5–18)	Score ≤ 5%^a^	84 (72)
*Platelet* ×10^3^/µL, mean value (range)	383 (110–998)	Score > 5%	32 (28)
*Splenomegaly*	68 (59)	*CVD at baseline*	
*Sokal score*		Positive anamnesis for CVD	23 (20)
Low	28 (24)	Myocardial infarction/angina	7 (6)
Intermediate	59 (51)	Arrhythmia	6 (5)
High	29 (25)	Other cardiac disease^b^	9 (8)
*Line of treatment*		Peripheral arterial disease	1 (1)
Second line	42 (36)	Stroke	1 (1)
Third line	47 (41)	Peripheral venous disease	0 (0)
Fourth line	27 (23)	*CV events following ponatinib*	
*Reason of switch*		Hypertension	15 (13)
Inefficacy	95 (82)	Myocardial infarction/angina	6 (5)
Intolerance	21 (18)	Peripheral arterial disease^c^	7 (6)
*Ponatinib dose at baseline*		Stroke	3 (3)
15 mg	18 (16)	*Primary prophylaxis*	19 (16)
30 mg	56 (48)	*Secondary prophylaxis*	6 (5)
45 mg	42 (36)	*Patient treated with statin/fibrate*	10 (9)
*Ponatinib dose at AOEs in 16 pts*		Atorvastatin	6 (5)
15 mg	5 (31)	Pravastatin	2 (2)
30 mg	6 (38)	Lovastatin	1 (1)
45 mg	5 (31)	Gemfibrozil	1 (1)

*CVD* cardiovascular disease, *AOEs* arterial occlusive events, *TKIs* tyrosine kinase inhibitors.

^a^The SCORE is based on the following risk factors: age, gender, smoking, systolic blood pressure and total cholesterol.

^b^Valvulopathy, restrictive cardiomyopathy, hypertensive cardiomyopathy.

^c^PAOD, atheromatic carotid disease.

At baseline, ponatinib was administered at the following doses: 45 mg/day in 36% of patients, 30 mg/day in 48% of patients, and 15 mg/day in 16% of patients, respectively. The median time of drug exposure was 16 months (range 1–60). In our cohort of 116 patients, 15 patients with hypertension and 16 with AOEs were recorded (Table 1). Among the patients with AOEs 31% had received 45 mg/day of ponatinib, 38% 30 mg/day and 31% 15 mg/day, respectively. No association was found between AOEs and dose of ponatinib or previous exposure to nilotinib. The median time elapsed between the start of ponatinib treatment and the onset of AOEs was 9 months (range 1–48). Overall, the 4-year cumulative incidence rate of AOEs was 26.5 ± 7%. Following the occurrence of AOEs and hypertension, 13 patients discontinued treatment; the dose of ponatinib was reduced in six patients and remained unchanged in 12. The 4-year cumulative incidence rate of MR4 following ponatinib treatment was 76.7 ± 11.7%, and it was not influenced significantly by AOE occurrence. Finally, the 4-year overall survival (OS) was 92.2 ± 3.4%.

Median plasma values and range of cholesterol, LDL, HDL, and triglycerides were collected at CML diagnosis, at the start of ponatinib therapy and after 3, 6, and 12 months of treatment. Triglycerides at the start of treatment with ponatinib, cholesterol, and LDL after 3 months of treatment were found significantly higher in comparison with others timepoints (*P* = 0.007, *P* = 0.02, and *P* = 0.002, respectively) (Supplementary Fig. 1).

**Fig. 1:**
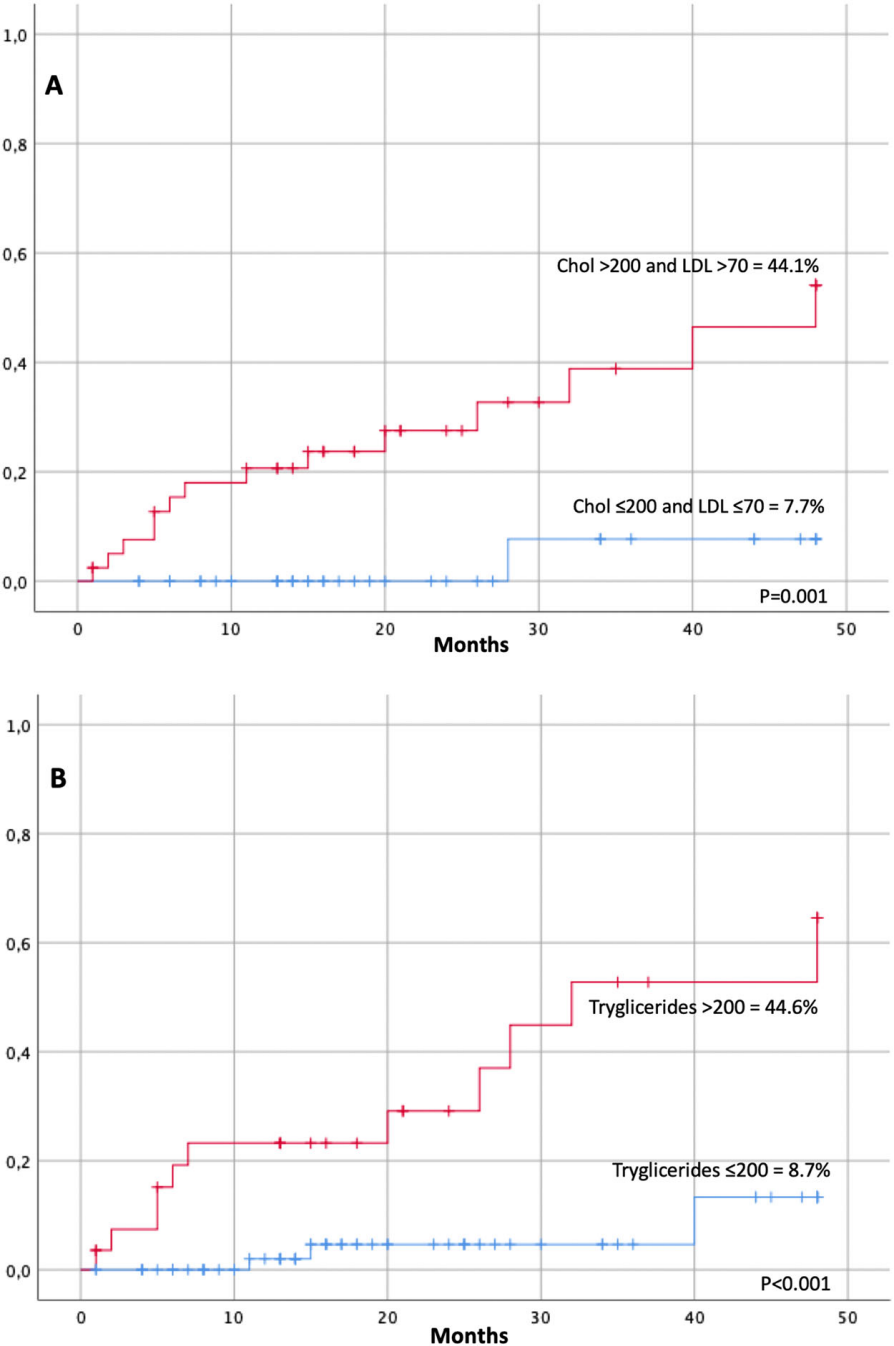
Arterial occlusive events (AOEs) in CML patients according to lipids levels. **a** AOEs in 82 CML patients according to cholesterol plasma level >200 mg/dL and LDL > 70 mg/dL after 3 months since starting ponatinib. **b** AOEs in 94 CML patients according to triglycerides plasma level >200 mg/dL before starting ponatinib. LDL low-density lipoprotein, Chol cholesterol.

Patients with cholesterol plasma levels > 200 mg/dL and LDL > 70 mg/dL after 3 months of treatment with ponatinib, showed a significantly higher incidence of AOEs (44.1 ± 11% vs. 7.7 ± 7.4, *P* = 0.001; data available on 82 patients) (Fig. 1a). The rate of AOEs was similar when considering at 3 months patients with a LDL threshold above 55 mg/dL. Patients with triglycerides plasma levels >200 mg/dL before starting ponatinib, showed a significantly higher incidence of AOEs (44.6 ± 14% vs. 8.7 ± 8.8, *P* < 0.001; data available on 94 patients) (Fig. 1b). In multivariate analysis, cholesterol plasma levels > 200 mg/dL and LDL > 70 mg/dL after 3 months and triglycerides plasma levels > 200 mg/dL before the start of ponatinib maintained a significant association with AOEs (*P* = 0.03; HR = 9.4; 95% CI = 1.2–72.5 and *P* = 0.004; HR = 7.1; 95% CI = 1.8–26, respectively).

Overall, 26 patients (22%) presented dyslipidemia at CML diagnosis and 41 (35%) at the start of treatment with ponatinib. Despite dyslipidemia, only 10 patients were taking statins during the treatment with ponatinib and only 2 started it after 3 months of ponatinib.

According to the new SCORE risk chart evaluation the majority of the 116 patients (72%) were classified at low to intermediate risk (SCORE risk ≤5%) and 28% of patients at high to very high risk (SCORE risk >5%). Patients belonging to the high and very high SCORE risk group showed a significantly higher incidence of AOEs (46.4 ± 15.3% vs. 20 ± 7.7%, *p* = 0.012) (Supplementary Fig. 2). In multivariate analysis the high–very-high SCORE risk maintained a significant association with AOEs (*p* = 0.04; HR = 2.9; 95% CI = 1–9.1).

Due to off-target effects, several adverse effects can occur in CML patients treated with TKIs, including endocrine and metabolic toxicity^9,10^. A possible role of TKIs as modifiers of the lipoprotein profile or in the atherogenic process has been scarcely explored. In vitro studies have shown that ponatinib elevates the levels of desmosterol, a substrate of 24-dehydrocholesterol reductase, the enzyme responsible for converting desmosterol to cholesterol^11^. In APOE*3Leiden.CEPT transgenic mice, a well-established model for dyslipidemia and atherosclerosis, it has been shown that nilotinib and ponatinib increased the CV risk through induction of a pro-thrombotic state^12^.

The role of lipoproteins as key initiating events in atherogenesis is becoming increasingly evident^7^. Small ApoB-containing lipoproteins can deposit within the arterial wall, causing a complex inflammatory process leading to lipid accumulation and formation of an atheromatic plaques It is an established fact that increased plasma concentrations of cholesterol-rich ApoB-containing lipoproteins are strongly associated to atherosclerotic CV disease and that lowering plasma LDL concentrations reduces CV events in humans^13^. We found that patients with triglycerides plasma levels >200 mg/dL before starting treatment with ponatinib and with cholesterol plasma levels >200 mg/dL and LDL > 70 mg/dL after 3 months from the start of ponatinib showed a significantly higher incidence of AOEs (Fig. 1). The rate of AOEs was similar considering at 3 months patients with a LDL threshold above 55 mg/dL. Moreover, we confirmed the predictive role of the modified SCORE risk system recently proposed^8^. The recent 2019 ESC/EAS guidelines for the management of dyslipidemia have highlighted the importance of lipid modifications to reduce the risk of CV events^8^. The authors recommend intervention strategies to keep the cholesterol and triglycerides values under 200 mg/dL and a therapeutic regimen that achieves a ≥50% LDL reduction from baseline and a target of LDL lower value of <70 mg/dL in high-risk patients and of <55 mg/dL in very-high patients. In our study, only 27% of patients belonged to the SCORE high and very-high risk group, but treatment with ponatinib could be considered “per se” a treatment potentially aggravated by atherothrombotic and CV complications that require a careful selection of patients. For this reason we suggest to consider patients aged ≥60 years or all patients with CVD, dyslipidemia, diabetes, or other CV risk factors before starting ponatinib, as “CV high risk” patients. These patients should be carefully investigated and monitored before and during ponatinib treatment and should maintain LDL values <70 mg/dL (Supplementary Table). Elevated plasma triglycerides values have been associated with an increased risk of atherothrombotic CV events. It has been suggested that the causal effect is determined by the circulating concentrations of ApoB-containing lipoproteins rather than by the triglyceride content^14^. ApoB analysis is now recommended for risk assessment, particularly in people with high triglycerides, diabetes, obesity, or metabolic syndrome^8^.

In order to reduce the risk of CV, in addition to change in lifestyle, a lipid-lowering therapy is recommended. In our real-life cohort of patients, at CML diagnosis 22% of them at diagnosis suffered from dyslipidemia that raised to 35% at the start of ponatinib treatment. Only 9% were assuming statins or fibrate, clearly indicating an under estimation of the clinical importance of elevated plasma lipids as a risk factor for CV events.

During treatment with ponatinib in CV low-intermediate risk patients, lipid values should be maintained within the normal range and specific lifestyle interventions on dietary habits and physical activity should be recommended. In patients at high risk of CV events, a lipid lowering statin-based therapy aimed at achieving LDL values <70 mg/dL should be suggested (Supplementary Table). If the goal is not reached with the maximum dose of statins, combinations with ezetimibe can be considered. In patients witnessing adverse events with statins, LDL lowering can be attempted with a different dose scheduling, such as every other day or twice weekly, with atorvastatin or rosuvastatin^15^.

Overall, personalized strategies to minimize the risk of AOEs should be thoroughly investigated in CML patients undergoing treatment with ponatinib; this could be particularly relevant for elderly patients with multiple comorbidities. These strategies include TKI interruption in patients with a stable MR4, since treatment-free remission (TFR) is now an emerging treatment goal for CML patients and several TFR predictive factors have been proposed^16,17^.

Despite the retrospective nature of the study, our findings emphasize that CML patients should be carefully investigated for their lipid profile at the start of ponatinib and during treatment in order to implement personalized prevention strategies based on a careful evaluation of the patient’s CV risk. Data on the efficacy of measures aimed at lowering LDL values <70 mg/dL in order to reduce AOEs need to be confirmed in larger cohorts of patients and in prospective randomized trials.

## Supplementary information

Supplemental figure 1

Supplemental figure 2

supplemental table

## Declarations

Data on patients were retrospectively collected in accordance with the 1975 guidelines of the Declaration of Helsinki.
