# Supplementary material for: Low low-density lipoprotein (LDL), cholesterol and triglycerides plasma levels are associated with reduced risk of arterial occlusive events in chronic myeloid leukemia patients treated with ponatinib in the real-life. A Campus CML study
Source: Blood Cancer J. 2020 Jun 8;10(6):66. doi: 10.1038/s41408-020-0333-2 (PMC7280258; doi:10.1038/s41408-020-0333-2)

**Supplemental Figure 1. Top: box plot diagram showing the distribution of lipoproteins levels during the patients’ follow-up**

In the simplest box plot the central rectangle spans the first quartile to the third quartile. A segment inside the rectangle shows the median and "whiskers" above and below the box show the locations of the minimum and maximum. The points are outliers, defined as values that do not fall in the inner fences. Outliers are extreme values. The asterisks are extreme outliers. These represent cases/rows that have values more than three times the height of the boxes.

**Bottom: median value and range of plasma cholesterol, LDL, HDL and triglycerides in CML patients at diagnosis, at the beginning of ponatinib and after 3, 6 and 12 months**

Cholesterol and LDL at 3 months after treatment and triglycerides at the start of treatment with ponatinib were found significantly higher in comparison with others timepoints

LDL= low-density lipoprotein; HDL= high-density lipoprotein; NS= not significant


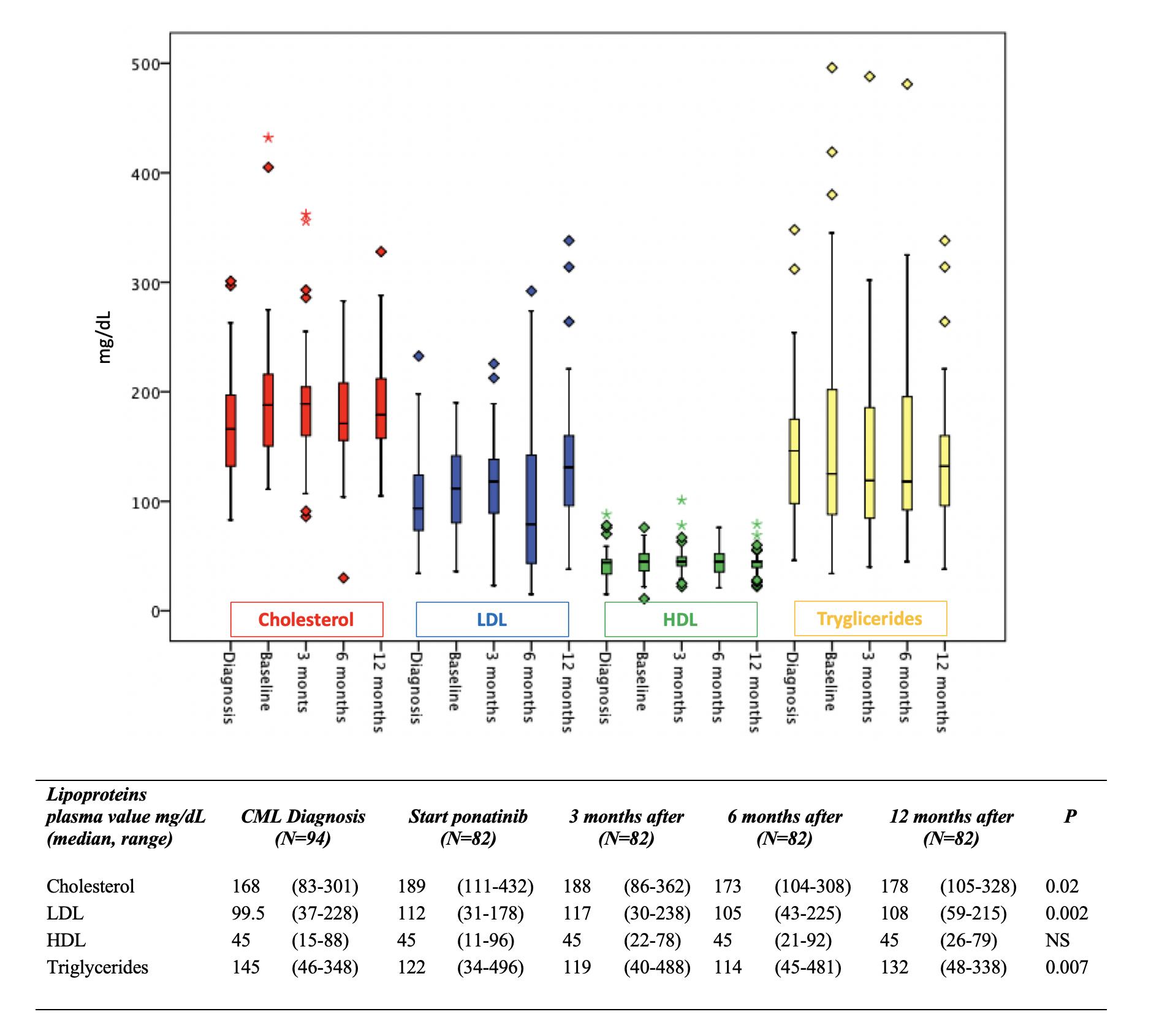

Supplement: Supplementary file 1 — Supplemental figure 1 [file 41408_2020_333_MOESM1_ESM.docx]
