# Supplementary material for: Low low-density lipoprotein (LDL), cholesterol and triglycerides plasma levels are associated with reduced risk of arterial occlusive events in chronic myeloid leukemia patients treated with ponatinib in the real-life. A Campus CML study
Source: Blood Cancer J. 2020 Jun 8;10(6):66. doi: 10.1038/s41408-020-0333-2 (PMC7280258; doi:10.1038/s41408-020-0333-2)
Supplement: Supplementary file 2 — Supplemental figure 2 [file 41408_2020_333_MOESM2_ESM.docx]

**
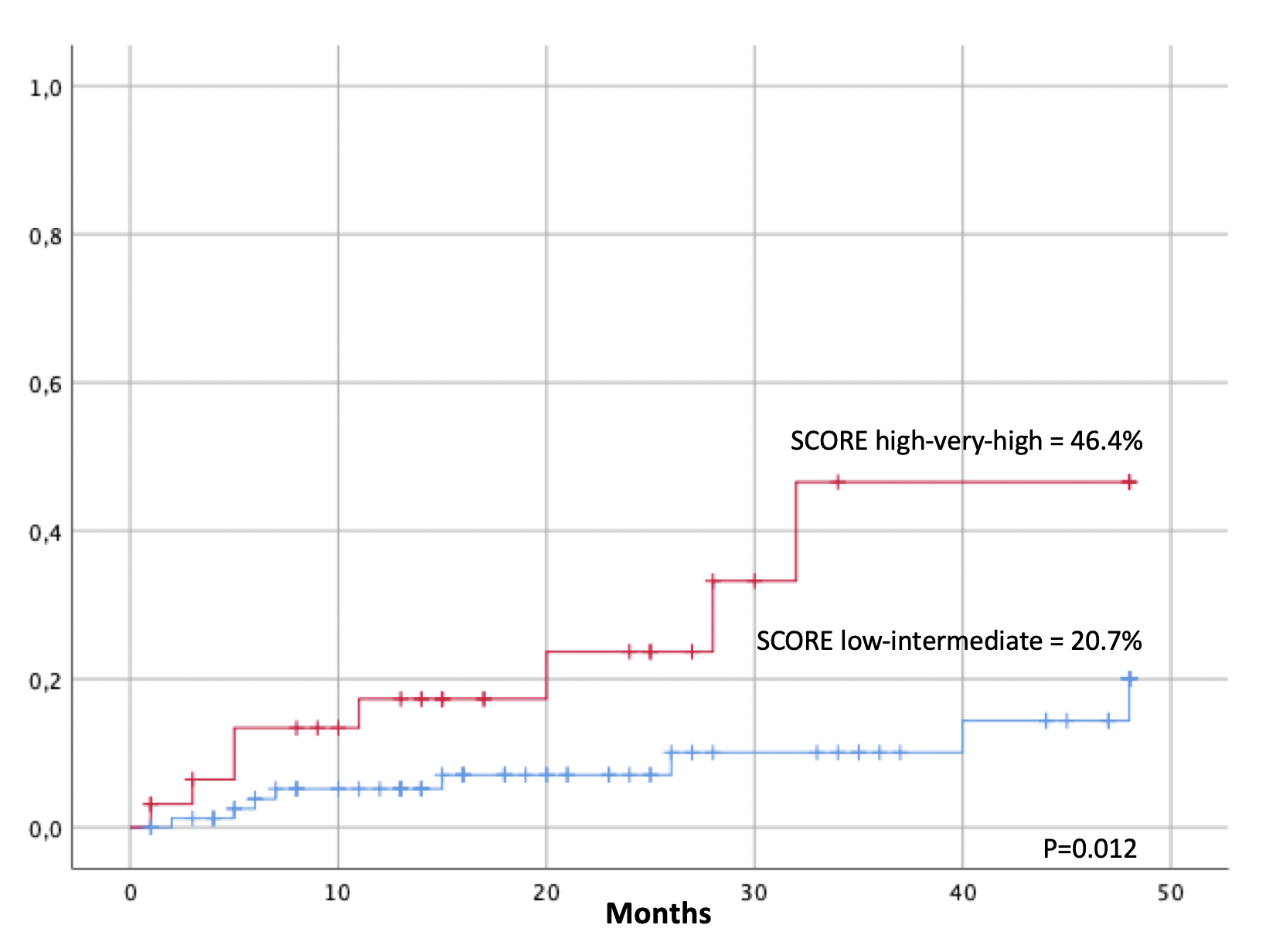
Supplemental Figure 2. Figure 4. Arterial occlusive events (AOEs) in 116 CML patients according to the SCORE risk**
