# Supplementary material for: Low low-density lipoprotein (LDL), cholesterol and triglycerides plasma levels are associated with reduced risk of arterial occlusive events in chronic myeloid leukemia patients treated with ponatinib in the real-life. A Campus CML study
Source: Blood Cancer J. 2020 Jun 8;10(6):66. doi: 10.1038/s41408-020-0333-2 (PMC7280258; doi:10.1038/s41408-020-0333-2)
Supplement: Supplementary file 3 — supplemental table [file 41408_2020_333_MOESM3_ESM.docx]

| **Evaluation of the cardiovascular risk, including the SCORE estimation and a complete lipid profile (cholesterol, LDL, HDL, triglycerides and, ideally, ApoB)** |
| --- |
| **In CV low-intermediate risk patients, lipid value should be maintained within normal range and specific lifestyle intervention on dietary habits and physical activity should be recommended** |
| **In CV high-very-high risk patients, or patients with diabetes or other CV risk factors or aged ≥ 60 years or presenting plaque burden on arterial ultrasonography, LDL value should be maintained < 70 mg/dL** |
| **In patients that do not reach the LDL target, the addition of a non-statin lipid-modifying agent such as ezetimibe to a maximally tolerated statin should be considered**  **In patients with adverse events by statin, LDL lowering can be obtained using different dose schedule, such as every other day or twice weekly, with atorvastatin or rosuvastatin**  **Prophylaxis with aspirin 100 mg/die should be considered in patients with CV risk factors, in particular if aged ≥ 60 years**  **Dose adjustment of Ponatinib in CV high-very-high should be considered** |

**Supplemental Table. Practical key issues in the management of dyslipidemia in CML patients starting ponatinib**

CV: cardiovascular; LDL: low-density lipoprotein; HDL: high-density lipoprotein
